# Immune evasion driven by lipid metabolic reprogramming in endocrine-resistant HR^+^ breast cancer: antigen presentation defects and T-cell dysfunction

**DOI:** 10.3389/fimmu.2026.1911296

**Published:** 2026-09-03

**Authors:** Lutian Gong, Yujing Zhao, Minglu Che, Xiangyang Zhang, Dongni Zhang, Wenping Lu

**Affiliations:** 1 Department of Oncology, China Academy of Chinese Medical Sciences Guang’anmen Hospital, Beijing, China; 2 Beijing University of Chinese Medicine, Beijing, China

**Keywords:** antigen presentation defects, endocrine-resistance, lipid metabolic reprogramming, T-cell dysfunction, hormone receptor-positive

## Abstract

Endocrine therapy has improved the prognosis of patients with hormone receptor-positive (HR^+^) breast cancer. However, acquired resistance remains a major cause of recurrence and progression, and the limited response of this subtype to immunotherapy highlights the need to better understand the mechanisms underlying immune escape. Recent studies indicate that, in addition to an immunosuppressive microenvironment, alterations in antigen processing and presentation may also contribute to immune escape, although systematic integration of these mechanisms remains lacking. Lipid metabolic reprogramming, as a key feature of tumor metabolic remodeling, supports tumor cell survival and adaptive evolution in endocrine-resistant HR^+^ breast cancer and may also regulate antitumor immune responses through multilevel mechanisms. At the tumor cell level, enhanced fatty acid oxidation, increased lipid synthesis, and altered cholesterol metabolism may affect tumor immune visibility by influencing antigen presentation-related processes, including MHC class I-associated pathways. Within the immune microenvironment, lipid accumulation may contribute to dendritic cell dysfunction and promote the polarization of tumor-associated macrophages toward immunosuppressive phenotypes, thereby impairing antigen presentation. Consequently, persistent and inefficient antigen stimulation may promote T-cell dysfunction and exhaustion. This article systematically integrates the role of lipid metabolism in antigen presentation and T-cell regulation, and proposes a dysfunctional multilevel mechanistic axis of “lipid metabolism-antigen presentation-T-cell function“, providing a theoretical basis for understanding immune evasion associated with endocrine resistance and for developing combination therapeutic strategies.

## 1 Introduction

Endocrine therapy has played an important role in improving the prognosis of patients with HR^+^ breast cancer (1, 2). However, acquired endocrine resistance remains relatively common and is closely associated with disease recurrence and progression (3). Although combination targeted therapies have, in recent years, delayed the onset of resistance to some extent, their long-term efficacy may still be influenced by tumor adaptive changes (4).

Meanwhile, immunotherapy has achieved significant progress in multiple solid tumors, but its efficacy in HR^+^ breast cancer remains relatively limited, particularly in endocrine resistance, where insufficient immune responses are often observed (5, 6). Existing studies suggest that, in addition to an immunosuppressive state, abnormalities in tumor antigen-related processes may also play an important role, including alterations in antigen expression, processing, and presentation (7, 8). However, current research has largely explored these mechanisms from individual perspectives, and a systematic understanding of their coordinated changes under resistant conditions remains lacking.

In recent years, metabolic reprogramming has gradually been recognized as playing an important role in tumor initiation, progression, and immune regulation. Among these, abnormalities in lipid metabolism have attracted increasing attention in HR^+^ breast cancer, particularly in the context of endocrine resistance (9, 10). Accumulating evidence indicates that metabolic reprogramming, particularly alterations in lipid metabolism, contributes to tumor cell survival, stress adaptation, and therapeutic responses (11–14). Meanwhile, immune cells within the tumor microenvironment can also undergo metabolic alterations, thereby affecting their functional states (11, 15). These findings suggest that lipid metabolism may serve as a potential link between tumor cells and the immune microenvironment. In addition, the functional state of T cells is a critical determinant of the effectiveness of antitumor immune responses (16). Existing studies have shown that under abnormal or persistently inefficient antigen stimulation, T cells may gradually develop a dysfunctional phenotype (17, 18). However, in endocrine-resistant HR^+^ breast cancer, the relationship between alterations in antigen-related processes and T-cell dysfunction remains to be further elucidated (19).

Therefore, this article focuses on lipid metabolic reprogramming, reviews its roles in tumor cells and immune cells, and summarizes current evidence regarding antigen presentation, tumor immune visibility, and T-cell function to provide a more systematic understanding of immune evasion in endocrine-resistant HR^+^ breast cancer.

## 2 Lipid metabolic reprogramming as the metabolic basis of endocrine-resistant HR^+^ breast cancer

Lipid metabolism refers to the collective biochemical processes involved in the synthesis, degradation, transport, utilization, and storage of lipid substances such as fatty acids, triglycerides, cholesterol, and phospholipids. It plays important roles in energy supply, membrane structure maintenance, signal transduction, hormone synthesis, and metabolic homeostasis (20, 21). Metabolic reprogramming is one of the important biological hallmarks that distinguishes tumor cells from normal cells. Under tumor microenvironmental stresses such as hypoxia, nutrient deprivation, inflammation, and activation of oncogenic signaling, tumor cells can adjust their metabolic patterns to meet survival and proliferative demands (22–24). Lipid metabolic reprogramming and tumor development are closely interconnected (26). On the one hand, tumorigenesis can disrupt normal lipid metabolic homeostasis, enabling tumor cells to acquire distinct lipid metabolic characteristics (25). On the other hand, lipid metabolic reprogramming may promote tumor progression and immune evasion by providing energy substrates and biosynthetic precursors, generating pro-tumorigenic metabolites, and shaping an immunosuppressive microenvironment, thereby contributing to tumor initiation and progression (27).

At the mechanistic level, tumor cells can coordinately enhance *de novo* fatty acid synthesis and fatty acid oxidation, while remodeling cholesterol biosynthesis pathways, thereby establishing a lipid metabolic adaptive network centered on energy supply, membrane structure maintenance, and signal transduction regulation (28–30). Tumor cells may activate the PI3K/AKT/mTOR signaling pathway, which in turn activates downstream sterol regulatory element-binding proteins (SREBPs), thereby upregulating lipid synthesis-related genes, including ACLY, ACC, and FASN, ultimately enhancing *de novo* lipogenesis (31–33). This process is supported by enhanced aerobic glycolysis in tumor cells, whereby pyruvate generated from glucose metabolism enters mitochondria to produce acetyl-CoA and is transported to the cytoplasm through citrate shuttling. ATP-citrate lyase (ACLY) generates cytoplasmic acetyl-CoA, which is subsequently used by FASN to synthesize long-chain fatty acids (34, 35). In addition, tumor cells can enhance exogenous fatty acid uptake by upregulating fatty acid translocase CD36 and fatty acid-binding proteins (FABPs), thereby compensating for limited endogenous synthesis (36, 37). Furthermore, fatty acid oxidation (FAO) can be enhanced by C-MYC activation and p53 functional loss, providing an alternative energy source and contributing to redox homeostasis (38). Regarding lipid storage, fatty acids can be esterified into triglycerides or cholesterol esters and stored in lipid droplets, serving as lipid reservoirs to cope with metabolic stress (39, 40).

In breast cancer, lipid metabolic abnormalities have been associated with endocrine resistance (41), and several studies have reported altered lipid metabolic profiles in endocrine-resistant HR^+^ breast cancer cells. For example, Sanna Hultsch et al. observed alterations in cholesterol metabolism and lipid droplet accumulation in tamoxifen-resistant cells (9). Ward et al. further reported increased FASN activity, enhanced *de novo* lipogenesis, and elevated triglyceride levels in resistant cells (42). These observations suggest that lipid metabolic reprogramming may contribute to the adaptive response of tumor cells to endocrine therapy, although whether it represents a driver or consequence of resistance remains to be further investigated.

Although current evidence does not fully establish a direct causal relationship between lipid metabolic reprogramming and endocrine resistance, increasing mechanistic studies suggest that these metabolic alterations may contribute to the emergence and maintenance of resistant phenotypes rather than merely represent secondary consequences of therapy exposure (43, 44). Endocrine treatment may impose metabolic selection pressure, under which tumor cells with enhanced lipid metabolic adaptability may gain survival advantages and become enriched within residual disease populations (45). Therefore, lipid metabolic reprogramming may function as both an adaptive response to endocrine therapy and a potential contributor to resistance development, providing a mechanistic rationale for discussing its role in maintaining resistant tumor states and shaping therapeutic vulnerabilities (28, 43).

## 3 Lipid metabolism-dependent adaptive states sustain the survival of therapy-resistant residual cells

Although endocrine therapy can inhibit the proliferation of HR-positive breast cancer cells, it rarely achieves complete tumor eradication. Persistent residual disease after treatment is considered an important cellular basis for late relapse and acquired resistance (46, 47). Phase III neoadjuvant endocrine therapy studies have shown that only approximately 20% of patients with ER-high/HER2-negative breast cancer achieve an endocrine-sensitive residual disease state, whereas most patients continue to harbor residual disease (48). Further molecular characterization has revealed that these residual cells retain survival capacity and display marked heterogeneity, including differences in PAM50 subtype switching and Ki-67 reduction (49). Therefore, residual cells are more likely to represent an adaptive cellular state shaped by continuous therapeutic pressure, rather than merely an inert cell population left behind because of incomplete treatment.

Accumulating evidence indicates that these residual cells may not simply represent a quiescent or dormant population (50, 51). Instead, they often exhibit enhanced stem-like properties and increased phenotypic plasticity, enabling transitions among dormant, reactivated, and invasive states and improving their adaptability to sustained therapeutic stress (52, 53). In this state, endocrine therapy-tolerant breast cancer cells exhibit increased dependence on oxidative phosphorylation (OXPHOS), and inhibition of this process can impair their survival and regrowth capacity in different models (45, 54). Liang et al. further demonstrated that ESR1 mutations and persistent therapeutic pressure can induce lineage plasticity in ER-positive breast cancer, leading to a mixed-lineage state and reduced dependence on standard endocrine therapy (55). Collectively, these findings suggest that residual cell maintenance is unlikely to depend on a single proliferative program, but rather involves coordinated remodeling of metabolic adaptability and cellular-state plasticity.

On this basis, lipid metabolic reprogramming, particularly the regulation of the FAO-OXPHOS metabolic axis, represents an important metabolic mechanism contributing to the maintenance of this adaptive state (43). Multi-omics studies have shown that the AMPK-FAO-OXPHOS pathway is activated in endocrine-resistant HR^+^ breast cancer. Targeting carnitine palmitoyltransferase 1 (CPT1) or inhibiting FAO has been reported to restore endocrine sensitivity and suppress the growth of resistant cells in certain models (43). Further mechanistic studies suggest that distinct upstream signaling pathways, including JNK/c-Jun-CPT1A-FAO, AMPK-ERRα/PGC-1β-MCAD/CPT1, FDXR-CPT1A-FAO, and PPARα-CPT1, may converge on FAO regulation through different regulatory levels, collectively contributing to a metabolic adaptive phenotype (44, 56–58). In addition, FAO may be functionally coupled with autophagy and multiple survival signaling networks, thereby supporting ATP production and cellular stress adaptation (43).

Beyond FAO, multilayered remodeling of lipid metabolic networks may also contribute to the maintenance of residual cell states. Evidence suggests that phospholipid remodeling may be involved in drug resistance development by affecting ER signaling stability (59). Triglyceride accumulation, increased lipid droplet formation, and enrichment of highly unsaturated lipids may help buffer fluctuations in energy metabolism and oxidative stress, thereby supporting the survival of residual cells under therapeutic pressure (42). ZMIZ1 lactylation promotes stemness enhancement and increased cholesterol uptake through the Nanog/NPC2 axis, which has been implicated in tamoxifen resistance (60). Meanwhile, the tumor microenvironment may also contribute to metabolic adaptation; for example, adipocyte-derived extracellular vesicles can enhance mitochondrial function in tumor cells and promote their transition toward OXPHOS dependence (61). Clinical observations further suggest that obesity, metabolic syndrome, and dyslipidemia are associated with altered responsiveness to endocrine therapy (10, 62).

## 4 Lipid metabolic reprogramming reshapes antigen presentation and tumor immune visibility

Lipid metabolic reprogramming is an important biological feature of endocrine-resistant HR^+^ breast cancer and is increasingly recognized as influencing immune-related phenotypes (42). First, the *de novo* fatty acid synthesis pathway is frequently activated, with key enzymes such as FASN being upregulated (42, 63). FASN catalyzes the synthesis of palmitate from acetyl-CoA and malonyl-CoA. These fatty acids not only serve as essential components of cellular membrane phospholipids, supporting membrane biosynthesis required for tumor cell proliferation, but may also influence tumor progression through signaling pathways such as PI3K/AKT/mTOR (64, 65). Second, the FAO pathway is activated, and resistant cells degrade fatty acids into acetyl-CoA through β-oxidation, while generating reducing equivalents (NADH and FADH2), thereby supporting mitochondrial ATP production (66). This metabolic flexibility enables resistant cells to maintain cellular functions under anti-estrogen conditions and enhances their adaptability to metabolic stress (57). In addition, cholesterol serves as a precursor for the synthesis of various signaling molecules and participates in regulating estrogen receptor activity and related oncogenic signaling pathways, which may facilitate adaptation of resistant cells to environmental changes (67). For example, cholesterol metabolites such as 25-HC and 27-HC can reactivate ER signaling and compensate for estrogen deprivation, thereby supporting sustained tumor cell growth (68). Collectively, these metabolic adaptations may contribute to the maintenance of endocrine-resistant states and influence tumor-immune interactions by altering cellular states and the tumor-immune interface.

On this basis, lipid metabolic reprogramming may participate in the regulation of antigen presentation and potentially reduce tumor immune visibility in HR^+^ breast cancer (69, 70). Previous studies have shown an inverse association between ER signaling and MHC-I expression, and HLA-I levels in HR^+^/luminal breast cancer are generally lower than those in basal-like subtypes, suggesting a relatively weaker antigen presentation capacity (71). At the transcriptional level, studies have found that under endocrine therapy or estrogen deprivation conditions, the expression of antigen presentation-related molecules such as β2-microglobulin (B2M) is downregulated, accompanied by suppression of the antigen presentation machinery, suggesting that endocrine adaptation may impair antigen processing and presentation pathways (72). In addition to transcriptional regulation, post-translational processes, including membrane localization, trafficking, and stability of MHC-I molecules, may also be affected (73). The membrane trafficking regulator MAL2, which is highly expressed in breast cancer, promotes endocytosis of peptide-loaded MHC-I complexes and their subsequent degradation in lysosomes, thereby reducing MHC-I surface availability and impairing CD8+ T-cell recognition and cytotoxic activity against tumor cells (74). Collectively, these findings indicate that impaired antigen presentation in HR^+^ breast cancer does not arise from a single defective step, but rather involves coordinated dysregulation of transcriptional control, intracellular trafficking, and membrane stability, which may ultimately reduce tumor immune visibility (75, 76).

Lipid metabolic abnormalities may influence tumor immune evasion by modulating antigen presentation-related processes, although the precise causal relationships and underlying mechanisms remain incompletely understood. At the tumor cell level, endocrine-resistant breast cancer is often characterized by enhanced fatty acid and cholesterol synthesis, altered membrane lipid composition, and lipid accumulation, which may influence MHC-I-related processes through multiple mechanisms (77, 78). On the one hand, MHC-I is distributed within cholesterol-enriched lipid rafts or distinct membrane microdomains, and its endocytosis is sensitive to cholesterol levels. Therefore, alterations in membrane lipid composition may influence MHC-I membrane localization, trafficking efficiency, and surface presentation. Studies have shown that MHC-I localizes to distinct plasma membrane environments and that its endocytic process is cholesterol-dependent, suggesting that the lipid environment may regulate its membrane trafficking (79). Other studies have found that DHA can alter lipid raft clustering and size, thereby affecting the lateral organization and surface availability of MHC-I (80). However, whether lipid metabolic reprogramming directly causes alterations in MHC-I expression and presentation remains to be further validated. On the other hand, increased lipid peroxidation may disrupt the structural integrity of membranous organelles such as the endoplasmic reticulum and induce endoplasmic reticulum stress responses, thereby interfering with MHC molecule folding, assembly, and antigen peptide loading and reducing peptide-MHC complex formation efficiency (81–83). In addition, transcriptional regulatory mechanisms may also contribute to these processes. Existing evidence suggests that estrogen receptor-associated transcriptional regulatory networks, such as the LCOR axis, are linked to antigen presentation programs, and disruption of this pathway may partially restore MHC-I expression and enhance T cell-mediated cytotoxicity (84). Meanwhile, key lipid metabolism-related transcription factors such as SREBP1 and SREBP2, together with endoplasmic reticulum stress-related molecules such as GRP78, may participate in immune regulation and potentially contribute to alterations in antigen presentation pathways and immune evasion-related phenotypes (85, 86).

## 5 Lipid metabolic reprogramming impairs antigen presentation in the tumor microenvironment

Current studies indicate that endocrine-resistant HR^+^ breast cancer cells may acquire metabolic adaptations by upregulating pathways such as *de novo* fatty acid synthesis, FAO, cholesterol metabolism, and lipid droplet accumulation (87, 88). Meanwhile, abnormal lipid accumulation in the tumor microenvironment may interfere with antigen presentation processes (89, 90), including impairing antigen uptake by dendritic cells (DCs) and modulating the polarization of tumor-associated macrophages (TAMs), thereby contributing to compromised anti-tumor immune responses and the occurrence of immune evasion (91).

DCs, as key antigen-presenting cells that initiate anti-tumor adaptive immunity, play a critical role in tumor antigen processing, cross-presentation, and priming of naive T cells (92). Research indicates that DC dysfunction may represent one mechanism through which lipid metabolic abnormalities in HR^+^ breast cancer influence antigen presentation capacity, potentially contributing to impaired antitumor immunity (93). Within a metabolically active, lipid-enriched TME, the accumulation of lipid droplets and oxidized lipids, increased levels of lipid peroxidation products, and persistent stimulation by tumour-derived factors may jointly enhance triglyceride synthesis in DCs (94, 95). This process may result in intracellular lipid accumulation, which has been associated with impaired antigen processing and cross-presentation capacity (96). During this process, DCs undergo metabolic remodeling. For instance, uptake of tumor-derived lipids (TDEs) can upregulate peroxisome proliferator-activated receptor α (PPARα) expression, thereby activating FAO and contributing to the formation of metabolically abnormal DCs, which may weaken their cross-presentation ability and limit effective T cell activation (97). Furthermore, the accumulation of oxidized lipids within DCs is considered a critical factor affecting antigen presentation. Evidence indicates that oxidized lipids, such as oxidized triglycerides, cholesterol esters, and fatty acids, can covalently interact with heat shock protein 70 (HSP70), thereby interfering with peptide-MHC loading and trafficking processes and inhibiting cross-presentation (98). Concurrently, the buildup of oxidized lipids in DCs may reduce surface availability of peptide-MHC class I (pMHC-I) complexes, impair cross-presentation of exogenous antigens (27, 99), and consequently affect downstream lymphocyte responses within the tumor tissue (100).

In the TME of HR^+^ breast cancer, lipid metabolic reprogramming, including upregulated expression of FABPs, lipoprotein lipase (LPL), and other lipid transport proteins, may promote the polarization of TAMs toward an M2-like immunosuppressive phenotype, thereby contributing to immune suppression and tumor progression (101–103). M2-like TAMs contribute to tumor immune suppression through multiple mechanisms. They secrete cytokines such as transforming growth factor-β (TGF-β) and interleukin-10 (IL-10) and express immune checkpoint molecules, thereby suppressing CD4^+^ and CD8^+^ T-cell function (104, 105). Moreover, M2-like TAMs exhibit reduced antigen-presenting capacity and decreased MHC-II expression, which may further impair T-cell activation (106, 107). Their secretion of IL-10 can further inhibit DC-mediated antigen presentation, thereby reducing T-cell activation and antitumor cytotoxic responses and contributing to immune evasion-related phenotypes (108). Beyond their antigen-presenting functions, TAMs regulate tumor and immune cell states through paracrine signaling and cell-cell communication. Through these mechanisms, TAMs can influence T-cell function through multiple pathways. For example, metabolites derived from M2-like TAMs, such as ceramides, can reshape immune inhibitory receptor programs and TREM2-associated macrophage states. In addition, TAM-T cell interactions through MHC-I-related signaling may promote the transition of T cells from progenitor exhaustion toward terminal exhaustion, thereby contributing to T-cell dysfunction within the tumor microenvironment (109, 110). These findings highlight the role of lipid-metabolically reprogrammed immune cells in shaping T-cell dysfunction within the tumor microenvironment.

## 6 Lipid metabolic reprogramming and persistent immune dysfunction promote T-cell exhaustion

Impaired antigen presentation and insufficient immune stimulatory signals may limit the ability of tumor-specific CD8^+^ T cells to receive sustained and effective antigenic stimulation, thereby contributing to progressive functional impairment and eventual exhaustion (84, 111, 112). Previous studies have shown that CD8^+^ T-cell antitumor responses depend on dendritic cell-mediated initial antigen presentation within tumor-draining lymph nodes, followed by effector differentiation (113). Furthermore, in endocrine-resistant HR-positive breast cancer, reduced MHC-I expression, impaired dendritic cell cross-presentation, and polarization of tumor-associated macrophages toward an immunosuppressive phenotype may collectively result in suboptimal and intermittent antigenic stimulation of CD8+ T cells, accompanied by enhanced inhibitory signaling, thereby creating a microenvironment conducive to the development of T-cell exhaustion (114, 115).

Against this background of imbalanced antigen presentation, regulatory T cells (Tregs) further reinforce defective immune priming through “co-stimulatory signal depletion” (116). In lipid-rich tumor microenvironments, Tregs exhibit enhanced utilization of exogenous lipids through fatty acid uptake, FAO activation, and mevalonate pathway activity. Through lipid transporters such as CD36, they increase metabolic adaptability, while PPAR signaling and FAO programs sustain Foxp3 stability and suppressive function (117, 118). Their intratumoral accumulation is driven by chemokines, including CCL22 (119). Mechanistically, Tregs can reduce the availability of CD80/CD86 costimulatory molecules on antigen-presenting cells (APCs) through a CTLA-4-dependent mechanism (120). This process impairs dendritic cell maturation and IL-12 secretion, decreases the expression of antigen-presenting and costimulatory molecules such as HLA-DR, CD40, CD80, CD86, and CD83, and consequently reduces the efficiency of T-cell activation (121–123). In addition, Tregs secrete inhibitory cytokines such as IL-10 and TGF-β and can indirectly sustain the immunosuppressive and lipid-metabolically altered state of TAMs (124). Consequently, Tregs may represent an important regulatory component linking impaired antigen-presentation capacity with reduced effector T-cell activation. Beyond these microenvironment-mediated effects, emerging evidence suggests that lipid metabolic alterations may also directly influence T-cell intrinsic metabolic programs and signaling pathways, thereby affecting T-cell persistence and effector functions.

Under persistent impairment of antigen presentation and costimulatory signaling, CD8^+^ T cells may gradually progress toward an exhaustion trajectory. Single-cell and spatial omics studies have revealed PD-1-high exhaustion-like CD8^+^ T-cell subsets in luminal breast cancer, accompanied by CXCL13 network remodeling and enhanced myeloid cell activation (125). Lineage analyses further indicate that the transition from PD-1Int to PD-1High CD8^+^ T cells may represent a continuous progression from a progenitor-exhausted state toward terminal exhaustion (126). Mechanistically, chronic exposure to insufficient or persistent antigenic stimulation can induce early effector dysfunction and epigenetic remodeling (127, 128). Such persistent stimulation may also promote p16 upregulation, suppress mTOR-associated metabolic programs, impair glycolysis and oxidative phosphorylation, and thereby accelerate the development of terminal exhaustion (129). Meanwhile, defective dendritic cell function, TAM-mediated TGF-β signaling, metabolic stress within the tumor microenvironment, and aberrant antigen presentation may further amplify this process, promoting CD8^+^ T-cell dysfunction within a milieu characterized by persistent insufficient stimulation and strong inhibitory signaling (130–132).

In addition to indirect regulation through lipid metabolic remodeling of the tumor microenvironment, emerging evidence suggests that abnormal lipid metabolism can directly influence T-cell intrinsic metabolic fitness, signaling activity, and functional maintenance. Within the tumor microenvironment, lipid-associated metabolic stress can impair CD8^+^ T-cell function and survival through multiple mechanisms. For example, PD-1 signaling in T cells can disrupt lipid homeostasis by suppressing PLPP1 expression, resulting in increased lipid peroxidation and ferroptosis susceptibility, thereby weakening antitumor activity (133). Increased acetyl-CoA carboxylase activity in T cells has been associated with abnormal lipid storage and lipid droplet accumulation, potentially impairing metabolic flexibility and long-term functional maintenance (134). Conversely, although cytotoxic T lymphocytes (CTLs) require fatty acid synthesis (FAS) and FAO to maintain energy supply and membrane homeostasis (135), dysregulated lipid metabolic pathways can alter T-cell functional states. Importantly, restoration of lipid metabolic balance may enhance antitumor immune responses. For instance, modulation of cholesterol metabolism within T cells demonstrates that lipid metabolic reprogramming can regulate T-cell signaling and effector function. Inhibition of the cholesterol esterification enzyme ACAT1 has been shown to increase plasma membrane cholesterol content, promote TCR clustering and downstream signal transduction, and enhance the effector function and proliferative capacity of CD8^+^ T cells (136). Nevertheless, most current evidence regarding lipid-mediated T-cell regulation has been obtained from preclinical models, and whether these metabolic dependencies are conserved in endocrine-resistant HR^+^ breast cancer requires further clinical investigation.

## 7 Lipid metabolic reprogramming mediated immune clearance impairment: a mechanistic axis involving defective antigen presentation and T-cell dysfunction

**Figure 1:**
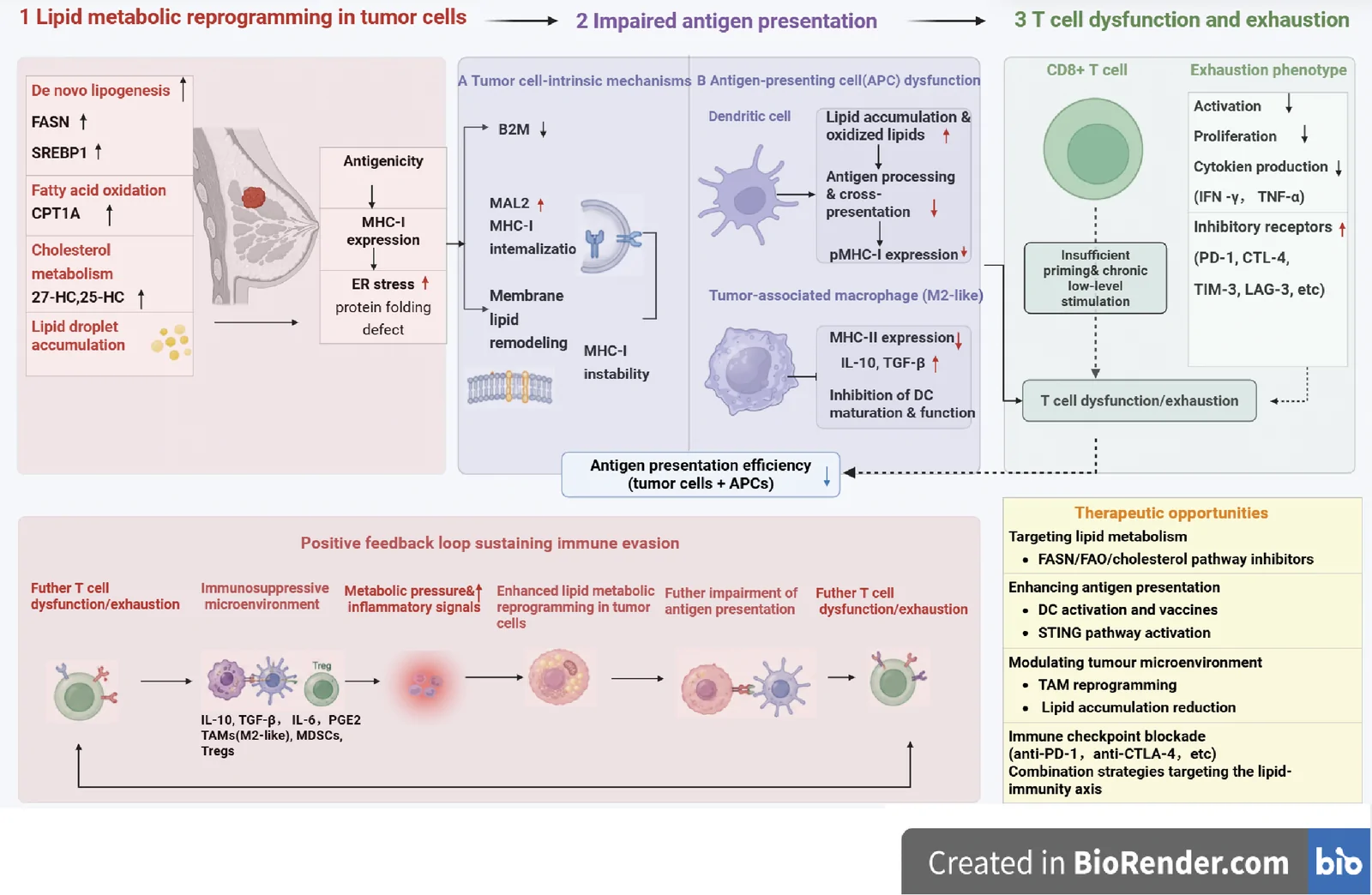
Lipid metabolic reprogramming contributes to immune evasion in endocrine-resistant HR^+^ breast cancer. Lipid metabolic reprogramming in endocrine-resistant HR^+^ breast cancer contributes to immune evasion through three interconnected processes: (1) metabolic adaptation of tumor cells reduces tumor immune visibility; (2) lipid accumulation and metabolic alterations in antigen-presenting cells impair antigen processing and presentation; (3) insufficient antigen stimulation contributes to T-cell dysfunction and exhaustion. These interconnected alterations establish an immunosuppressive tumor microenvironment and highlight potential therapeutic strategies targeting metabolic-immune interactions. (Figure was created using https://app.biorender.com/).

In summary, lipid metabolic reprogramming may play a cross-level regulatory role in endocrine resistance and immune evasion in HR^+^ breast cancer. Its impact extends beyond metabolic adaptation of tumor cells and may involve reshaping the initiation and amplification processes of antitumor immune responses, thereby contributing to impaired immune surveillance (32). From a systems-level perspective, lipid metabolic abnormalities may reduce tumor immune visibility by impairing antigen presentation efficiency, thereby altering the threshold for immune recognition and limiting effective initiation of antitumor responses (77). At the same time, this metabolic environment may further enhance the strength of immunosuppressive signaling, thereby inhibiting effective T-cell activation and limiting their functional expansion (137). Therefore, lipid metabolic reprogramming may contribute to the construction of a dynamic immunosuppressive network characterized by reduced tumor immune visibility, impaired immune priming, and compromised T-cell activation, ultimately weakening antitumor immune responses at the system level (138). This framework suggests that lipid metabolism is not only a consequence of tumor metabolic adaptation but may also represent one of the important upstream regulatory factors driving immune evasion associated with endocrine resistance (**Figure 1**).

## 8 Therapeutic strategies and clinical implications of targeting lipid metabolic reprogramming

Based on the close association between lipid metabolic reprogramming, endocrine resistance, impaired antigen presentation, reduced tumor immunogenicity, and T-cell dysfunction, therapeutic strategies for endocrine-resistant HR^+^ breast cancer need to extend beyond simple ER signaling inhibition toward an integrated approach that simultaneously considers metabolic regulation, immune restoration, and endocrine responsiveness (139). Clinical translation of this strategy, however, requires careful consideration of tumor metabolic plasticity, immune context, and patient heterogeneity, as metabolic intervention, immune sensitization, and endocrine resensitization represent biologically interconnected rather than independent therapeutic processes.

### 8.1 Targeting lipid metabolic reprogramming: from suppressing resistant cell survival to restoring immune sensitivity

Accumulating evidence suggests that FASN, carnitine palmitoyltransferase 1A (CPT1A)-mediated FAO, and the cholesterol-mevalonate pathway are involved in endocrine therapy resistance in HR^+^ breast cancer and may also influence immunosuppressive phenotypes (43, 45, 140, 141). The therapeutic significance of targeting lipid metabolism has expanded beyond merely inhibiting tumor proliferation toward restoring metabolic vulnerability and immune sensitivity. Among these, FASN, as a key rate-limiting enzyme in fatty acid synthesis, is associated with downregulation of MHC-I-related molecules, adaptive upregulation of PD-L1, and impaired cytotoxic immune responses, suggesting its critical role in linking metabolism to immunosuppression. Inhibition of FASN can reduce tumor cell resistance to T cell-mediated killing and may enhance sensitivity to anti-PD-1 therapy (140, 141). On the other hand, the FAO pathway serves as an important metabolic foundation for endocrine resistance and the maintenance of residual cells. Sustained activation of the AMPK-FAO-OXPHOS axis in HR^+^ breast cancer suggests that CPT1A/FAO may support metabolic adaptation of tolerant residual cells and may be associated with immune evasion (43, 45). CPT1A-mediated FAO has been implicated in reducing tumor cell susceptibility to immune-mediated cytotoxicity, and its inhibition may provide a potential strategy for simultaneously disrupting metabolic adaptation and enhancing immune sensitivity (43). In addition, the cholesterol metabolism-related mevalonate pathway is also associated with breast cancer progression and therapy resistance. Clinical and epidemiological studies suggest that statins may have potential value in reducing recurrence risk and improving prognosis (142, 143), potentially through mechanisms such as downregulating PD-L1 expression and enhancing CD8^+^ T cell function (144, 145). Therefore, statins may modulate immune evasion through multiple mechanisms, although their clinical translational value remains to be further validated. Despite these encouraging findings, several challenges remain for the clinical translation of lipid-targeted therapies. Cancer cells can undergo metabolic adaptation, and lipid metabolic pathways are also essential for normal immune cell function, potentially limiting the efficacy and specificity of systemic metabolic targeting (146).

### 8.2 Immunotherapy and antigen presentation enhancement: key strategies to increase tumor immune visibility

From an immunotherapeutic perspective, HR^+/^HER2^-^ breast cancer generally exhibits limited responsiveness to PD-1/PD-L1 blockade, which may be associated with relatively low tumour immune visibility, a myeloid-dominated immunosuppressive tumour microenvironment, and insufficient antigen presentation (147). Studies have suggested that immune checkpoint inhibitors may provide selective benefits in subgroups with high PD-L1 expression or abundant tumor-infiltrating lymphocytes (TILs), although overall efficacy remains limited (147). Given the critical role of impaired antigen presentation in immune evasion of endocrine-resistant HR^+^ breast cancer, merely releasing PD-1/PD-L1-mediated inhibitory signals may be insufficient to restore effective tumor-specific T cell responses. Therefore, strategies aimed at antigen presentation enhancement or DC reconstruction may provide important complementary value (84). Nevertheless, enhancing antigen presentation alone may not be sufficient to overcome immune resistance because effective antitumor immunity also requires functional T cells and a permissive tumor microenvironment (148). Although direct evidence in breast cancer remains relatively limited, existing studies have suggested that HER2-targeted type 1 DC vaccines, intratumoral injection of cDC1, and antigen-specific DC vaccines can enhance local T cell infiltration and antigen-specific immune responses to some extent (149–152). Meanwhile, STING pathway activation contributes to cDC1 maturation and initial CD8^+^ T cell activation, indicating its potential value in improving the “immune cold” tumor state (153). However, the therapeutic application of STING activation requires further optimization because excessive immune stimulation may lead to inflammatory toxicity or limited durability (154). Based on these observations, metabolic intervention and antigen presentation enhancement may be viewed as complementary rather than independent therapeutic approaches, as they have the potential to increase tumor immune visibility and create a more favorable context for subsequent immune checkpoint blockade, potentially facilitating endocrine resensitization.

### 8.3 Combination treatment strategies

Consistent with this integrated therapeutic framework, therapeutic strategies may need to expand beyond single ER signaling inhibition toward a multi-axis approach integrating metabolic, immune, and endocrine regulation. Combining metabolic intervention with immunotherapy, such as PD-1/PD-L1 blockade, may provide opportunities to enhance T cell cytotoxic function and achieve potential metabolic-immune synergy. Clinical studies have shown that pembrolizumab combined with aromatase inhibitors exhibits limited efficacy in metastatic HR^+^ breast cancer, suggesting that endocrine therapy alone combined with immune checkpoint inhibitors may be insufficient to overcome the “immune cold” tumor state (155). In contrast, in premenopausal patients with ER^+/^HER- advanced breast cancer, PD-1 blockade combined with endocrine therapy has demonstrated more pronounced immune activation signals (156). In early high-risk populations, neoadjuvant immunotherapy has also been shown to improve pathological response rates, indicating that tumor burden and immune plasticity may influence therapeutic efficacy (157). Furthermore, strategies combining CDK4/6 inhibitors or epigenetic modulators with immune checkpoint inhibitors suggest that remodeling the immune microenvironment could potentially expand the window of opportunity for immunotherapy, although the balance between therapeutic benefit and toxicity remains to be fully elucidated (158, 159). A biologically rational strategy may involve targeting lipid metabolic reprogramming to disrupt metabolic adaptations that sustain endocrine resistance while simultaneously enhancing antigen presentation and increasing tumor immune visibility (42, 112). These metabolic changes may subsequently promote immune sensitization by facilitating T-cell activation and remodeling the immunosuppressive tumor microenvironment (32, 93). Enhanced antitumor immunity may subsequently contribute to the control of therapy-resistant tumor cell populations and potentially improve endocrine responsiveness, supporting a sequential therapeutic framework of metabolic intervention, immune sensitization, and endocrine resensitization rather than a simple combination of individual treatment modalities (160, 161). Despite promising preclinical evidence, the optimal combination partners, treatment sequence, and biomarker-guided patient selection strategies remain to be defined, particularly in the context of metabolic heterogeneity across endocrine-resistant HR^+^ breast cancer (162). This framework provides a mechanistic basis for developing biomarker-guided precision metabolic-immune-endocrine therapeutic strategies (163). A comprehensive summary of the current therapeutic strategies targeting lipid metabolic reprogramming and immune dysfunction, including their biological rationale, potential advantages, translational limitations, and candidate biomarker-guided patient selection strategies, is provided in **Supplementary Table 1**.

### 8.4 Biomarker-guided patient selection for precision metabolic-immune therapy

Beyond identifying effective therapeutic combinations, a major challenge for clinical translation is determining which patients are most likely to benefit from metabolic-immune therapeutic strategies (42, 164). Endocrine-resistant HR^+^ breast cancer exhibits substantial inter-tumor and intra-tumor heterogeneity in lipid metabolic states and immune microenvironmental features (28, 165). Consequently, the efficacy of metabolic-immune combination strategies may vary across patients, highlighting the need for biomarker-guided patient stratification and precision therapeutic approaches (166).

Tumor-intrinsic metabolic characteristics may provide a promising basis for patient selection. Candidate biomarkers, including FASN expression, CPT1A/FAO-related metabolic signatures, cholesterol-mevalonate pathway activation, and lipid droplet accumulation, may reflect distinct metabolic dependencies and potential therapeutic vulnerabilities (42, 167). Importantly, these metabolic phenotypes may not be uniform across endocrine-resistant HR^+^ breast cancer and may vary according to molecular subtype (e.g., luminal A versus luminal B), acquired resistance mechanisms (e.g., ESR1-mutant versus ESR1-wild-type tumors), and previous therapeutic exposure (168, 169). Such biological heterogeneity may influence both lipid metabolic dependency and immune responsiveness, thereby affecting the efficacy of metabolic-immune combination strategies (170).

In addition to tumor-intrinsic metabolic features, systemic metabolic parameters, such as serum lipid profiles, represent attractive and readily accessible complementary biomarkers (10). However, their clinical utility may be limited by insufficient tumor specificity and the influence of host metabolic status (170). Furthermore, intra-tumoral metabolic heterogeneity may lead to the coexistence of metabolically distinct cellular populations within the same tumor, potentially contributing to differential therapeutic responses and adaptive resistance following metabolic intervention (171).

Given the complexity of metabolic heterogeneity and tumor-immune interactions, a single biomarker is unlikely to be sufficient for accurately predicting therapeutic responsiveness (164). Instead, integrating lipid metabolic characteristics with immune-related parameters, including antigen presentation capacity, PD-L1 expression, and T-cell infiltration, may provide a more robust framework for precision patient selection and rational design of metabolic-immune combination therapies (172). Nevertheless, these candidate biomarkers remain largely investigational and require prospective clinical validation across distinct molecular and metabolic subgroups before they can be incorporated into routine clinical decision-making.

## 9 Conclusion

Overall, lipid metabolic reprogramming represents an important link between endocrine resistance and immune evasion in HR^+^ breast cancer. By affecting tumor immune visibility, the antigen presentation system, and immune cell functional states, it may contribute to the formation of a metabolism-associated immunosuppressive network, leading to impaired T-cell activation and exhaustion. However, the causal relationships and dynamic mechanisms among lipid metabolism, antigen presentation abnormalities, and T-cell dysfunction remain to be further elucidated. Future studies integrating multi-omics and spatially resolved technologies may help identify key regulatory nodes and evaluate the potential of lipid-targeted strategies combined with immunotherapy and endocrine therapy to overcome resistance and improve therapeutic efficacy.
