# Supplementary material for: Immune evasion driven by lipid metabolic reprogramming in endocrine-resistant HR+ breast cancer: antigen presentation defects and T-cell dysfunction
Source: Front Immunol. 2026 Sep 3;17:1911296. doi: 10.3389/fimmu.2026.1911296 (PMC13582620; doi:10.3389/fimmu.2026.1911296)
Supplement: Supplementary file 1 [file Table1.docx]

**Supplementary Table S1.** Therapeutic strategies, candidate biomarkers, and translational considerations for metabolic–immune therapy in endocrine-resistant HR^+^ breast cancer

| **Strategy** | **Biological rationale** | **Candidate biomarkers** | **Potential advantages** | **Current limitations** |
| --- | --- | --- | --- | --- |
| FASN inhibition | FASN-driven lipogenesis supports tumor metabolic adaptation and may contribute to immune suppressive phenotypes^[42]^ | FASN expression (IHC); lipogenic gene signatures^[162]^ | May reduce lipid synthesis and potentially improve tumor immune visibility | Limited clinical validation; metabolic compensation; possible effects on normal lipid metabolism and immune cells |
| CPT1A/FAO inhibition | FAO supports survival of endocrine-resistant residual cells and may contribute to immune dysfunction^[38,43]^ | CPT1A expression; FAO/OXPHOS-related signatures ^[167]^ | May target metabolic vulnerabilities and potentially improve immune responsiveness | FAO plasticity; potential effects on immune cell metabolism; lack of clinically validated inhibitors |
| Cholesterol-mevalonate pathway inhibition (statins) | Cholesterol metabolism contributes to tumor progression and immune modulation^[67,68,98]^ | cholesterol pathway activation; SREBP-related signatures; systemic lipid profiles as exploratory complementary markers ^[9,42]^ | Clinically available drugs with potential immunomodulatory effects | Evidence mainly retrospective/preclinical; unclear causal benefit in HR^+^ breast cancer |
| Lipid storage/metabolic vulnerability targeting | Lipid droplet accumulation reflects altered lipid handling and metabolic adaptation | Lipid droplet content; lipid storage-related signatures | May identify tumors with altered lipid storage and metabolic adaptation | Detection methods are not standardized; limited clinical evidence |
| DC-based antigen enhancement strategies | Enhance antigen presentation and facilitate T-cell priming^[150-152]^ | antigen presentation-related signatures; cDC1-related signatures^[40,149-152]^ | Increase tumor immune visibility | Limited efficacy when used alone due to persistent T-cell dysfunction and immunosuppressive TME |
| STING pathway activation | Promote innate immune sensing and cDC1-mediated CD8+ T-cell activation^[153]^ | STING pathway activity; immune-inflamed phenotype ^[153]^ | Potential to enhance immune infiltration | Toxicity, adaptive resistance, and context-dependent responses |
| Immune checkpoint blockade and combination strategies | Reinvigorate dysfunctional/exhausted T-cell responses^[156-159]^ | Integrated lipid-immune signatures combining metabolic features (e.g., FASN/FAO-related signatures) with immune parameters (e.g., PD-L1, TILs, antigen presentation) | Potential synergy with metabolic remodeling | Limited efficacy in HR^+^ disease; optimal combination and sequencing remain unclear |

Note: The references cited in the table are the same as those cited in the main text.
